# Intercomparison of MODIS AQUA and VIIRS I-Band Fires and Emissions in an Agricultural Landscape—Implications for Air Pollution Research

**DOI:** 10.3390/rs10070978

**Published:** 2018-06-21

**Authors:** Krishna Vadrevu, Kristofer Lasko

**Affiliations:** 1 NASA Marshall Space Flight Center, Huntsville, AL 35812, USA; 2 Department of Geographical Sciences, University of Maryland, College Park, MD 20740, USA; klasko@umd.edu

**Keywords:** remote sensing, emissions, crop residue burning, active fires, fire radiative power

## Abstract

Quantifying emissions from crop residue burning is crucial as it is a significant source of air pollution. In this study, we first compared the fire products from two different sensors, the Visible Infrared Imaging Radiometer Suite (VIIRS) 375 m active fire product (VNP14IMG) and Moderate Resolution Imaging Spectroradiometer (MODIS) 1 km fire product (MCD14ML) in an agricultural landscape, Punjab, India. We then performed an intercomparison of three different approaches for estimating total particulate matter (TPM) emissions which includes the fire radiative power (FRP) based approach using VIIRS and MODIS data, the Global Fire Emissions Database (GFED) burnt area emissions and a bottom-up emissions approach involving agricultural census data. Results revealed that VIIRS detected fires were higher by a factor of 4.8 compared to MODIS Aqua and Terra sensors. Further, VIIRS detected fires were higher by a factor of 6.5 than Aqua. The mean monthly MODIS Aqua FRP was found to be higher than the VIIRS FRP; however, the sum of FRP from VIIRS was higher than MODIS data due to the large number of fires detected by the VIIRS. Besides, the VIIRS sum of FRP was 2.5 times more than the MODIS sum of FRP. MODIS and VIIRS monthly FRP data were found to be strongly correlated (r^2^ = 0.98). The bottom-up approach suggested TPM emissions in the range of 88.19–91.19 Gg compared to 42.0–61.71 Gg, 42.59–58.75 Gg and 93.98–111.72 Gg using the GFED, MODIS FRP, and VIIRS FRP based approaches, respectively. Of the different approaches, VIIRS FRP TPM emissions were highest. Since VIIRS data are only available since 2012 compared to MODIS Aqua data which have been available since May 2002, a prediction model combining MODIS and VIIRS FRP was derived to obtain potential TPM emissions from 2003–2016. The results suggested a range of 2.56–63.66 (Gg) TPM emissions per month, with the highest crop residue emissions during November of each year. Our results on TPM emissions for seasonality matched the ground-based data from the literature. As a mitigation option, stringent policy measures are recommended to curtail agricultural residue burning in the study area.

## 1. Introduction

In several regions of the world, biomass burning is one of the significant sources of atmospheric aerosols and trace gas emissions, which have a significant impact on climate and human health [1–3]. In addition to forest biomass burning, crop residue burning is prevalent in several countries of Asia [4–9]. In India, the key agricultural residues generated (in million metric tons, MMT) include rice straw (112), rice husk (22.4), wheat straw (109.9), sugarcane tops (97.8) and bagasse (101.3), most of which are burnt in open air [2]. Several studies have shown smoke particles from biomass burning impacting radiative forcing by scattering and absorbing shortwave radiation and indirect radiative forcing by serving as cloud-condensation nuclei (CCN) and changing the cloud microphysical and optical properties [10–12]. The magnitude and signs of aerosol radiative forcing are mainly determined by the scattering and absorption characteristics of aerosols released during biomass burning [13,14]. Specifically, crop residue burning has been reported to emit CO, non-methane hydrocarbons (NMHC), NO_x_, SO_2_, particulate matter and other greenhouse gases (CO_2_, N_2_O, and CH_4_) [15–17]. Also, crop residue burning and biomass burning pollution, in general, is known to cause severe health effects such as chronic obstructive pulmonary disease (COPD), pneumoconiosis, pulmonary tuberculosis, bronchitis, cataract, corneal opacity and blindness [18,19]. Further, crop residue burning releases a significant amount of smoke which can reduce visibility, thus causing road accidents [2,20].

In South Asia, the Indo Gangetic Plains (IGP) straddling the northeastern parts of India near the foothills of the Himalayas is one of the most densely populated regions in the world, with substantial anthropogenic emissions. In addition to factories and vehicular traffic, agricultural crop residue burning is one of the major contributors to pollution in the region [21–25]. Mostly, farmers burn agricultural residues during the summer (April–May) and winter (October–November) each year in the IGP, which has a significant impact on greenhouse gas emissions and aerosol loading [3,15]. The IGP in India covers 20% of the geographical area, contributes 42% to the total food grain production and holds nearly 40% of the total population [21]. In India, the IGP includes Punjab, Haryana and western Uttar Pradesh which are mostly dominated by agriculture [3,21]. Rice-wheat crop rotation is commonly practiced in the IGP. Mostly, farmers use combines for harvesting wheat during summer and rice during the winter season. Combines leave behind large quantities of residues in the field which is difficult to remove; thus farmers burn the residues. Specifically, in Punjab, earlier studies on estimating emissions from crop residue burning have used agricultural production data, a crop specific residue-to-product ratio, an estimate of the proportion of residue subject to burning, emission factors, and a combustion factor [23–25].

Compared to agricultural production-based emission estimates, use of remote sensing technology for quantifying fires and the resulting emissions has a huge potential [26–29]. Satellite remote sensing technology with its multi-spectral, multi-temporal, synoptic and repetitive coverage can provide useful information on the fire cycle, type of vegetation burnt, amount of vegetation burnt including intensities [29–37]. Currently, three different fire products are derived from the polar and geostationary satellite sensors: (a) active fires which can be detected through the elevated thermal radiance signal typical of flaming and smoldering conditions [36–38]; (b) burned area product derived mostly through measuring the changes in surface reflectance before and after the fire from red, near infrared and short-wave infrared channels [39,40] and (c) fire radiative power (FRP) products based on the measured rate of radiant energy output of the detected fires [28,33,35]. Using these fire products, earlier researchers quantified the emissions from different landscapes, including forests, savannas, grasslands and agricultural fires [38,41]. In addition to Moderate Resolution Imaging Spectroradiometer (MODIS) (http://modis-fire.umd.edu/index.php), fire products from Visible Infrared Imaging Radiometer Suite (VIIRS) data (http://VIIRSfire.geog.umd.edu/) are readily available. While the potential of remote sensing data for fire detection and estimating the resulting emissions is well recognized, recent research suggests significant under-detection of burnt areas in the agricultural landscapes [37]. Also, sensor differences, including resolution, along-scan aggregation, and swath width can result in differences in fire detection as in the case of MODIS and VIIRS fire detection [37]. Some of the methods to mitigate fire emissions underestimation due to clouds, view angles and swath gaps can be found in the literature [42,43].

Thus, there is a strong need to evaluate different fire products, especially in agricultural regions and landscapes to assess their potential and limitations for emissions estimation. The analysis needs to be region-specific and source-specific to generate useful emission estimates to enable emissions mitigation. In this study, we focus on the Punjab state in the IGP where agricultural residue burning is prevalent, for quantifying emissions using both the MODIS and VIIRS satellite-derived fire datasets. We address the following questions.

How does the MODIS (MCD14ML) fire product compare with the VIIRS (VNP14IMG) fire product in the agricultural landscape of Punjab, India? What are the seasonal differences? How does FRP vary between MODIS Aqua and VIIRS (VNP14IMG) satellite data? What are the implications of using MODIS versus VIIRS FRP data for emissions estimation? How do bottom-up emission estimates integrating crop production data compare with satellite-derived Global Fire Emissions Database (GFED) emissions, as well as MODIS and VIIRS FRP based emission estimates? We answered the above questions by integrating ground-based crop production data, both regional and general emission factors and satellite data (MODIS and VIIRS). The results provide valuable insights into crop burning related emissions in Punjab, India.

## 2. Materials and Methods

### 2.1. Study Area

**Figure 1.**
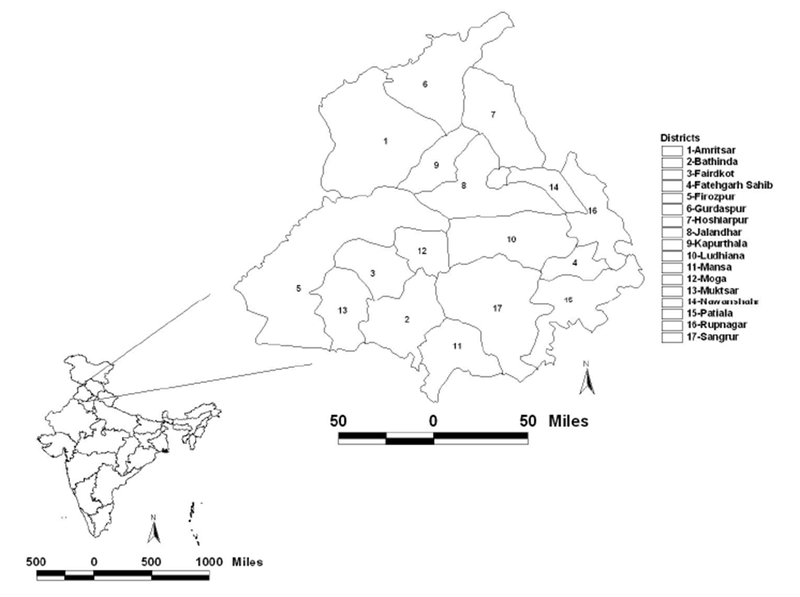
Study area location map depicting Punjab state in India with different districts.

**Figure 2.**
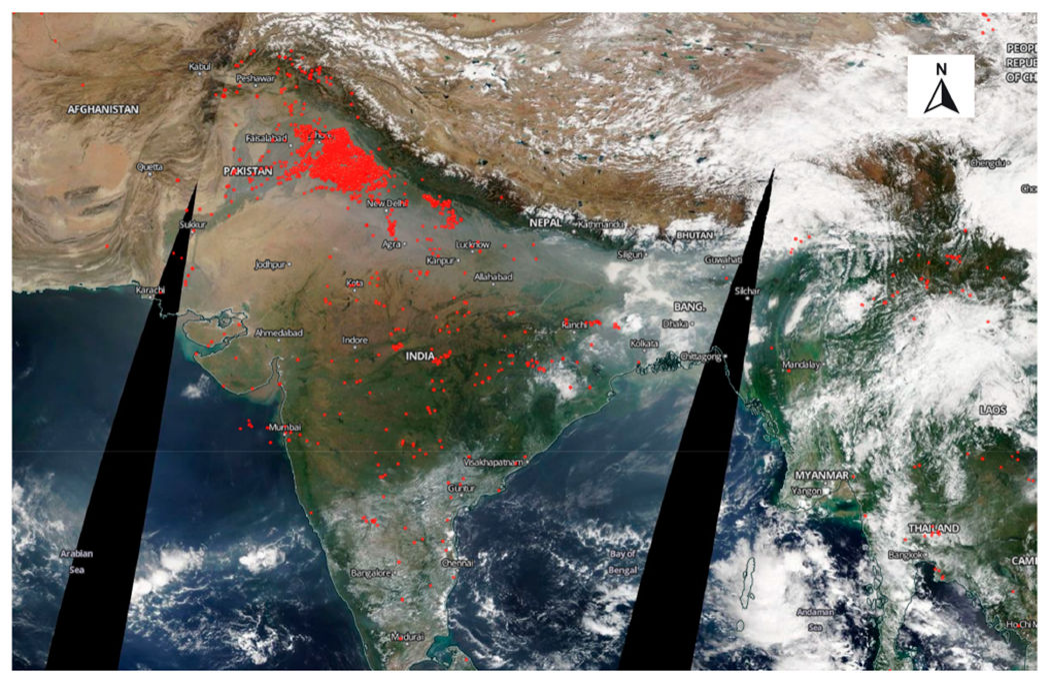
Agricultural fires (in red) occurring in Punjab, India retrieved from the Visible Infrared Imaging Radiometer Suite (VIIRS) on 25 October 2017 during crop residue burning (Source: NASA WorldView).

Punjab is a state in northern India (Figure 1) with border states of Jammu and Kashmir to the north, Himachal Pradesh to the east, Haryana to the south and southeast, Rajasthan to the southwest, and the Pakistan province of Punjab to the west (https://en.wikipedia.org/wiki/Punjab,India). The total geographical area of the state is 5.036 million hectares (ha) of which the cultivable area is 4.20 million ha and the net area sown during 2015–2016 was 4.138 million ha (http://agripb.gov.in). Ninety-nine percent of the net sown area is under irrigated agriculture through canals, tube wells and other water sources (http://agripb.gov.in). On average, rice (37.15%) and wheat (48.76%) together constitute 85.91% of grown crops. Crops such as maize, jowar, cotton, and others constitute only 14% of the total cropped area (http://agripb.gov.in/home.php?page=astat). In this region, rice is usually grown in the wet summer season (sown in July–August and harvested in October–November) and wheat in the dry winter season (sown in November–December and harvested in April–May). The summer wheat residue burning season is during April and May, and winter rice residue burning is during October and November. The agricultural fires captured by the Suomi NPP VIIRS data on 25 October 2017 in Punjab, northwest India is shown in Figure 2. More details about the seasonal characteristics of crop residue burning and causative factors are given in [32].

In addition to local pollution, the residue burning in Punjab and Haryana has been causing an immense pollution problem in the capital city of India, New Delhi which lies close to the above two states [44]. Although, both the Haryana and Punjab governments have imposed a ban on burning of paddy residues, farmers still practice residue burning as the time gap between Rabi planting, and Kharif harvesting is two to three weeks; thus, burning of residue is the fastest way to clear the land for the winter crop. The removal of paddy stalks after harvesting is labor-intensive; thus, farmers prefer burning the residues [3,15]. However, the residue burning releases smoke and aerosol particles leading to adverse air quality and health risks [18–21].

### 2.2. Emissions Estimation Using Agricultural Census Data

We calculated the maximum potential emissions from crop residue burning based on the following equation [21,24,25,45]:

(1) $$\mathrm{ECBR}=\sum_{\text{Crops}}^{\text{n}}A\times B\times C\times D\times E\times F$$

where ECBR is the emissions from crop residue burning (in Gg), A is the total crop production (in million tonnes), B is the residue to product ratio, C is the dry matter fraction, D is the fraction burnt and E is the fraction oxidized and F is the emission factor for total particulate matter (TPM). The following crops were considered for ECBR: upland rice, paddy rice, wheat, sugarcane, cotton, Kharif (monsoon) grown pulses, Rabi (winter) oil seeds and Kharif (monsoon) oilseeds. We gathered the crop production values from the office of the Directorate of Agriculture, Government of Punjab, India statistical division. Of the total production, rice accounts for 31.92%, paddy for 21.39%, wheat for 30.37%, sugarcane for 10.98%, rabi oilseeds for 0.10%, Kharif oilseeds for 0.006%, sunflowers for 0.178%, and total Kharif pulses for 0.017%. The residue to product ratios used were as follows, rice: 1.50; wheat 1.70; maize 1.50; sugarcane: 0.40; cotton: 3.0; oilseeds: 2.2; pulses: 1.50 [23–25,45]. Dry matter fraction has been estimated as eighty percent of the total production and the fraction oxidized as eighty percent following the IPCC 1996 guidelines [46]. The emission factor for total particulate matter (TPM) emission rates for agricultural residues in Asia vary from 4.53–8.75 g/kg for different types of residues burnt [8]. In this study, we used a mean of 6.64 g/kg while estimating the TPM emissions using the ground-based dry matter burnt approach as well as GFED emissions.

### 2.3. VIIRS I-Band Fire Product

The first VIIRS was launched in October 2011 aboard the Suomi-National Polar-orbiting Partnership (S-NPP) satellite. The VIIRS instrument carries two separate sets of multi-spectral channels providing full global coverage at both 375 m and 750 m nominal resolutions every 12 h or less depending on the latitude. The VIIRS satellite incorporates fire-sensitive channels, including a dual-gain, high-saturation temperature 4 μm channel, enabling active fire detection and characterization. The active fire product, based on the 375 m (I-bands) and 750 m moderate resolution “M” bands of VIIRS, are currently generated [37]. In this study, we specifically used the VIIRS 375 m active fire product (VNP14IMG). The algorithm for this product builds on the well-established MODIS Fire and Thermal Anomalies product using a contextual approach to detect thermal anomalies [37]. Due to its higher spatial resolution, the VNP14IMG active fire product captures more fire pixels than the MODIS MCDML product [37,47]. Specific to the FRP, the VNP14IMG FRP is calculated through a combination of both VIIRS 375 m and 750 m data. The former is used to identify fire-affected, cloud (solid blue), water (dashed blue), and valid background pixels. Then, co-located M13 channel radiance data (750 m) coinciding with fire pixels and valid background pixels are used in the FRP calculation. More details about the product can be found in [37].

### 2.4. MODIS Active Fire Products

We used the latest collection of 6.1 MODIS active fire products (MCD14ML) from the University of Maryland College Park (http://modis-fire.umd.edu/index.php). The active fire dataset is at a 1 km nominal spatial resolution; however, it can detect flaming fires up to 50 m^2^ under ideal observation conditions [34,47]. The MODIS fire product provides the latitude and longitude of the fire pixels, the date and time of the fire detection, and its fire radiative power (FRP). The FRP is a quantitative measure of radiant heat output commonly used to approximate fire intensity, which is proportional to its combustion rate and smoke emissions [29,30,33,35,48,49].

### 2.5. Fire Radiative Power Products and Emissions

Fire Radiative Power (FRP) is the rate of fire energy released per unit time, measured in megawatts [33,35]. Fire radiative energy (FRE) is therefore, FRP integrated over time and space with units in mega joules. The MODIS FRP (MW) is calculated using the Wooster et al. 2003 approach [33] in which FRP is approximated as

(2) $$\mathrm{FRP}\approx \frac{A_{\mathrm{pix}}\sigma }{a\tau_{4}}(L_{4}-{\bar{L}}_{4})$$

where *L*_4_ is the 4 μm radiance of the fire pixel, ${\bar{L}}_{4}$ is the 4 μm background radiance, *A*_*pix*_ is the area of the MODIS pixel (which varies as a function of scan angle), *σ* is the Stefan-Boltzmann constant (5.6704 × 10^−8^ W m^−2^ K^−4^), *τ*_4_ is the atmospheric transmittance of the 4 μm channel, and *a* is a sensor-specific empirical constant. For MODIS, *a* = 3.0 × 10^−9^ W m^−2^ sr^−1^ μm^−1^ K^−4^ when radiance is expressed in units of W m^−2^ sr^−1^ μm^−1^ [47].

For the VIIRS 375 m (VNP14IMG) product, 375 m mid-IR (I4) radiance data for FRP retrieval is not used due to frequent data saturation/folding. Quality flags (QF1) assigned during L1B onboard data aggregation will not indicate partial saturation. Instead, FRP is retrieved using co-located dual-gain mid-IR M13 channel (750 m) for all fire pixels detected using 375 m data and then divided by two to get 375 m FRP [37].

FRP measurements have been previously related to the amount of biomass burnt [33,35], the strength of fires [28] and aerosol emissions [50–53]. The FRE based emission coefficients for quantifying the gas and aerosol emissions from biomass burning have been developed by [33] from field experiments and by [48] from laboratory measurements. Further, References [51–53] demonstrated the utility of a an FRE based approach to quantifying biomass burning emissions of organic and black carbon aerosols.

References [33,53] inferred that mass of smoke aerosol released from biomass burning can be linearly related to FRE. Reference [53] related the rate of aerosol emission (R_osa_ in kg/s) to FRP as:

(3) $$R_{\mathrm{osa}}=C_{e}*\mathrm{FRP}$$

where C_e_ is the coefficient that directly relates radiative power from fire to its smoke aerosol emission rate (coefficients of emission in kg/MJ for particulate matter) and mass of smoke aerosol emission (M_esa_) as:

(4) $$M_{\mathrm{esa}}=C_{e}*\mathrm{FRE}$$

Using the above approach, Reference [53] developed a global emissions inventory product entitled “Fire Energetics and Emissions Research (FEER)” based on collocated satellite FRP and aerosol optical thickness (AOT) observations. Inferred total particulate matter emission rates are linked to observe FRP. The estimated TPM emission coefficients allow direct conversion from time-integrated FRP to emitted particulate matter without invoking the emission factors [53].

In this study, using the above approach from [53], we derived the TPM emissions from agricultural residue burning in Punjab using MODIS (2003–2016) and VIIRS (2012–2016) datasets. Specifically, we used only the FEER coefficient (kg/MJ) of TPM emission and not the total FEER inventory as we focused on a small study area dominated by agricultural fires in India. The FEER TPM coefficients fall within 0.01 and 0.1 kg/MJ for different land cover type related fires, and specifically for agricultural landscapes, we used a value of 0.04 kg/MJ [53]. Since geostationary data was lacking to capture diurnal fire cycles in the study region, we used the sum of FRP to arrive at the mass of smoke aerosol emissions. We used MODIS as well as VIIRS FRP to characterize the differences in TPM emissions for different years, for comparative purpose. Besides, we also compared the emissions derived from the dry matter burnt approach and GFED emissions for TPM emissions.

### 2.6. GFED Emissions Product

The Global Fire Emission Database (GFED) [54] comprises emissions from grassland, savanna, and forest fires, including deforestation fires. In the GFED, emissions are calculated as the product of MODIS burned area, estimated dry fuel loads derived from satellite net primary productivity, combustion completeness, and generalized land cover emission factors [54,55]. Satellite-derived burned areas drive the fire module of a CASA biogeochemical model that calculates fuel loads for each month and grid cell. Combustion completeness is also calculated in the model based on fuel types and moisture conditions. The latest version of GFED (Version 4s) has a spatial resolution of 0.25 degrees and is available from 1997-present. In this version, the burned area from “small” fires based on active fire detection outside the burned area maps are also included [http://www.globalfiredata.org/data.html]. In this study, we used the GFED data from 2003 onwards and used the particulate matter emission factor from [16] to estimate the emissions for Punjab from 2012–2016.

### 2.7. Temporal Emissions Integrating MODIS and VIIRS

VIIRS data is available only from 2012, whereas MODIS Aqua data is from May 2002-present; thus, to account for the VIIRS temporal FRP-based emissions prior to 2012, a prediction model with the peak Gaussian fit has been derived with MODIS Sum of FRP as the regressor (x) variable and VIIRS Sum of FRP as the response (y) variable. The resulting model is given as:

(5) $$\mathrm{aExp}\left(-\left(\frac{1}{2}{\left(\frac{\left(\mathrm{MODISFRPSUM}-b\right)}{c}\right)}^{2}\right)\right)$$

where *a* is the peak value; *b* is the critical point and *c* is the growth rate. For developing the model, we partitioned seventy percent of the original data into training data and used the remaining thirty percent for validating the model. Using the model parameters and MODIS sum of FRP from the testing dataset, we derived the VIIRS sum of FRP values. We then compared the latter values with the true VIIRS sum of FRP from the testing dataset. Also, we used a mean absolute percentage error (MAE) statistic as a measure of model performance as

(6) $$\mathrm{MAE}=\frac{1}{N}*\sum_{k=1}^{N}|t_{k}-y_{k}|$$

where, *y_k_* and *t_k_* denote the model output and measured value from the *k*th element; $\bar{y}$ and $\bar{z}$ denote their average respectively, and *N* represents the number of observations. The peak FRP value was found to be 352,075.2, with a critical point of 1,696,447.6 and growth rate of 67,796.4 and the overall model fit had the highest r^2^ of 0.93. Using these parameters, we derived the TPM emissions from 2003–2016.

## 3. Results

### 3.1. MODIS versus VIIRS Fire Counts

**Figure 3.**
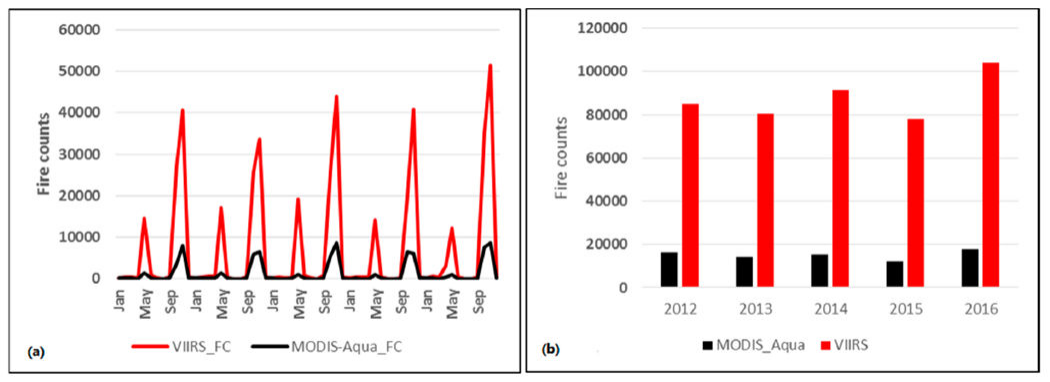
(**a**) Monthly fire counts for Punjab, India derived from VIIRS versus MODIS (AQUA and TERRA combined) data and for different years; (**b**) Averaged across five years, VIIRS detected fires were higher by a factor of 4.8 than MODIS data.

**Figure 4.**
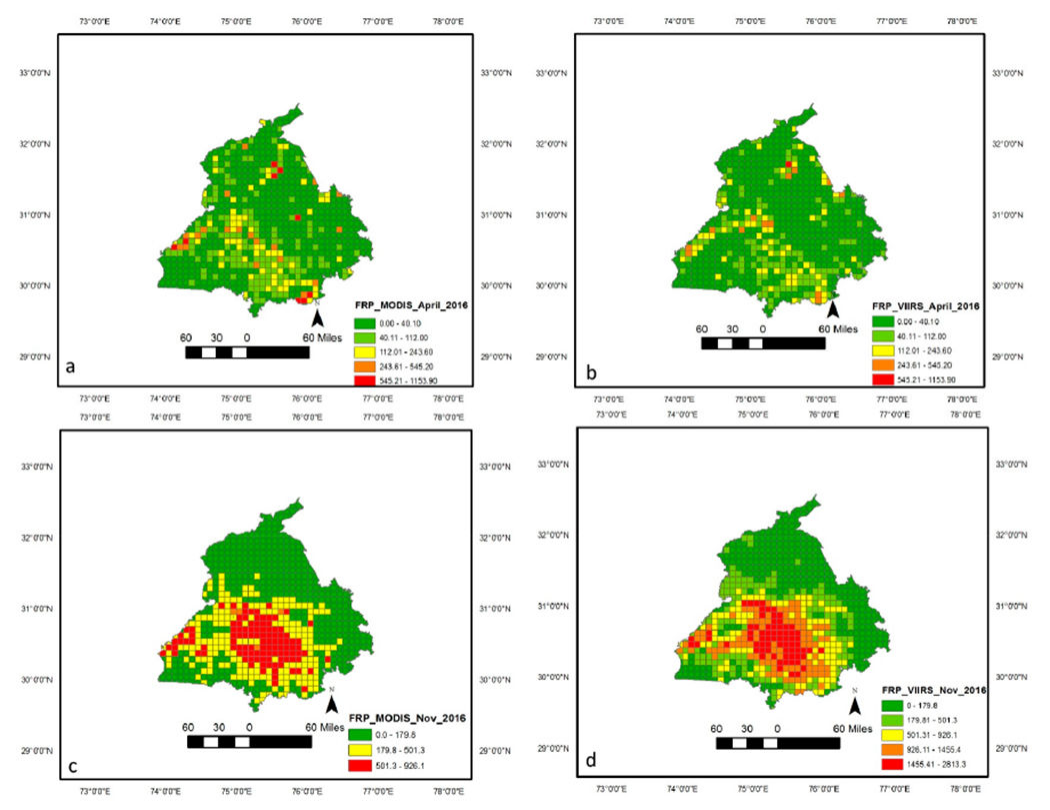
Sum of fire radiative power (FRP) from MODIS and VIIRS for the peak months of summer (April) (**a,b**) and winter (November) (**c,d**), 2016. FRP data has been gridded at 5 arc minute grid intervals (10 km cells). High FRP during November captured by VIIRS data can be seen in Figure 4d.

Averaged across fourteen years (2003–2016), the combined MODIS Aqua and Terra fire counts for Punjab were found to be 18,270 (σ = 2521). In contrast, fire counts from VIIRS data averaged across five years (2012–2016) suggested 87,702 fire counts (σ = 10,352), almost 4.8 times higher than the MODIS data (Figure 3a,b). Further, mean MODIS Aqua fire counts (2003–2016) were found to be 13,489 compared to mean VIIRS fire counts (2012–2016) of 87,702, the latter being higher by a factor of 6.5 than Aqua. Further, the mean monthly MODIS Aqua FRP was found to be 16.10 (MW) compared to the mean VIIRS FRP of 5.25 (MW). The sum of fire radiative power (FRP) from MODIS and VIIRS for the 2016 peak months of summer (April) and winter (November) are shown in Figure 4a,b and Figure 4c,d, respectively. The annual sum of VIIRS FRP was found to be 2.25 times more than the annual sum of MODIS FRP. For example, averaged across five years (2012–2016), the annual sum of FRP for VIIRS was 622,420 MW compared to the MODIS annual sum of FRP 276,000 MW.

**Figure 5.**
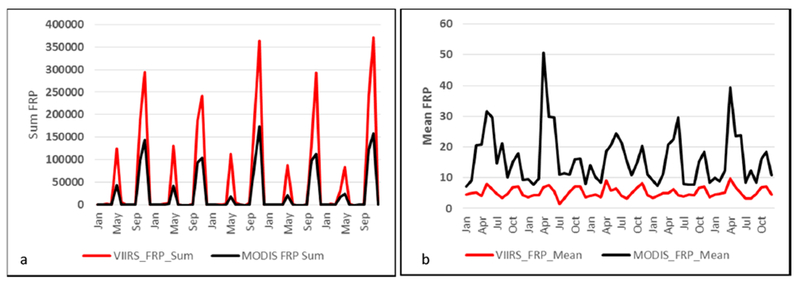
Seasonal variations in monthly sum of fire radiative power (FRP) (**a**) and mean FRP (**b**) for Punjab, India derived from MODIS (Aqua) and VIIRS data. Note that the mean MODIS Aqua FRP is higher than the VIIRS FRP, whereas the sum of FRP is higher for VIIRS than MODIS due to the relatively large number of fires detected by VIIRS.

**Figure 6.**
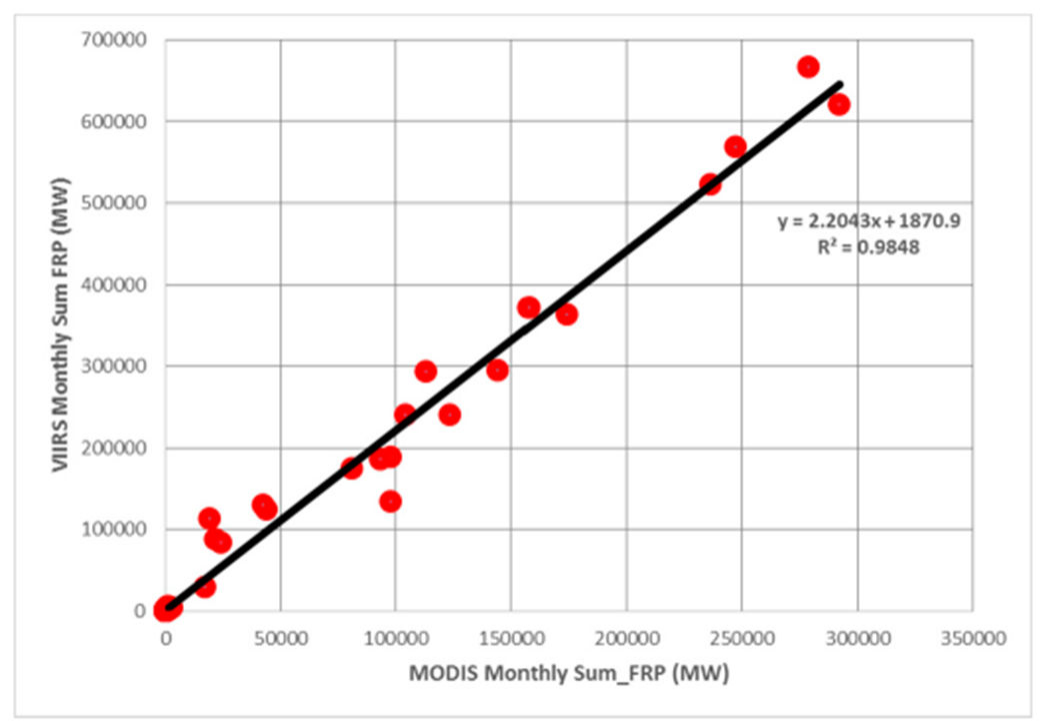
Correlation between MODIS Sum of FRP and VIIRS Sum of FRP, Punjab, India.

FRP data has been gridded at five arc minute grid intervals (10 km cells). Higher FRP during November captured by VIIRS data can be seen in the Figure 4d. Seasonal variations in the monthly sum of FRP and mean FRP are shown in Figure 5a,b. The mean monthly MODIS Aqua FRP was found to be 16.10 (MW) compared to mean VIIRS FRP of 5.25 (MW) (Figure 5b). However, the sum of FRP from VIIRS data was found to be higher than the MODIS sum of FRP (Figure 4b), mainly due to the large number of fires detected by VIIRS. Since the VIIRS FRP and the MODIS AQUA FRP are retrieved using the 4.0 μm channel and both cross the equator at approximately the same time (1:30 a.m. descending and 1:30 p.m. ascending) local time, a strong correlation between FRP data retrieved from these two datasets is expected. In our study area, which is dominated by agricultural fires, we observed a strong correlation coefficient (r^2^ = 0.98) (Figure 6).

### 3.2. Agricultural Census Data Based Emissions

Using the agricultural census data from 2005–2016, the total carbon burnt for the study area has been estimated to be in the range of 10.45–11.04 Tg per year. Most of the residues were found to be from upland rice, paddy rice, wheat and sugarcane which contributed 94.66% of the total carbon burnt. From these data, the total TPM emissions were estimated to be 88.19–91.19 Gg using the bottom-up approach from the agricultural census data.

### 3.3. GFED Emissions

Results from the GFED data suggested an average of 6.09–7.31 Tg dry carbon of residues burnt per year for the study region. These values are considerably lower than the bottom-up estimate using the agricultural census data, similar to an earlier comparison study in Vietnam [56]. Analysis of GFED data suggested October, November, and December with the highest contribution to the total carbon emissions. Further, the TPM emissions were found to be in the range of 42.0–61.71 Gg for the study region which is lower than the bottom-up estimate.

### 3.4. FRP-Based Emissions

**Figure 7.**
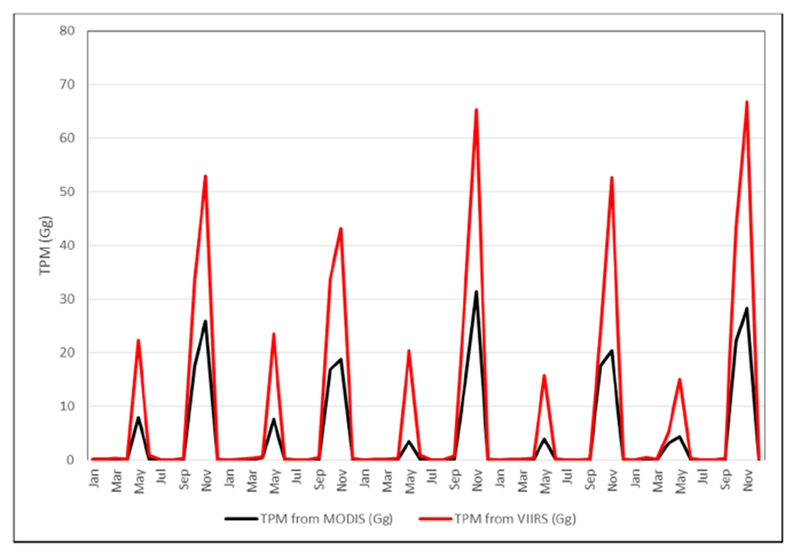
Total Particulate Matter (TPM) emissions in Gg derived from MODIS and VIIRS data.

**Figure 8.**
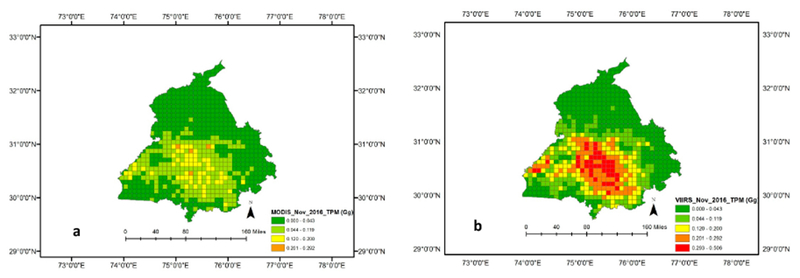
TPM emissions (in Gg) from MODIS (**a**) and VIIRS for the peak month of November (**b**), 2016. TPM emissions data has been gridded at 5 arc minute grid intervals (10 km cells). High FRP during November captured by VIIRS data can be seen in the Figure 8b.

**Figure 9.**
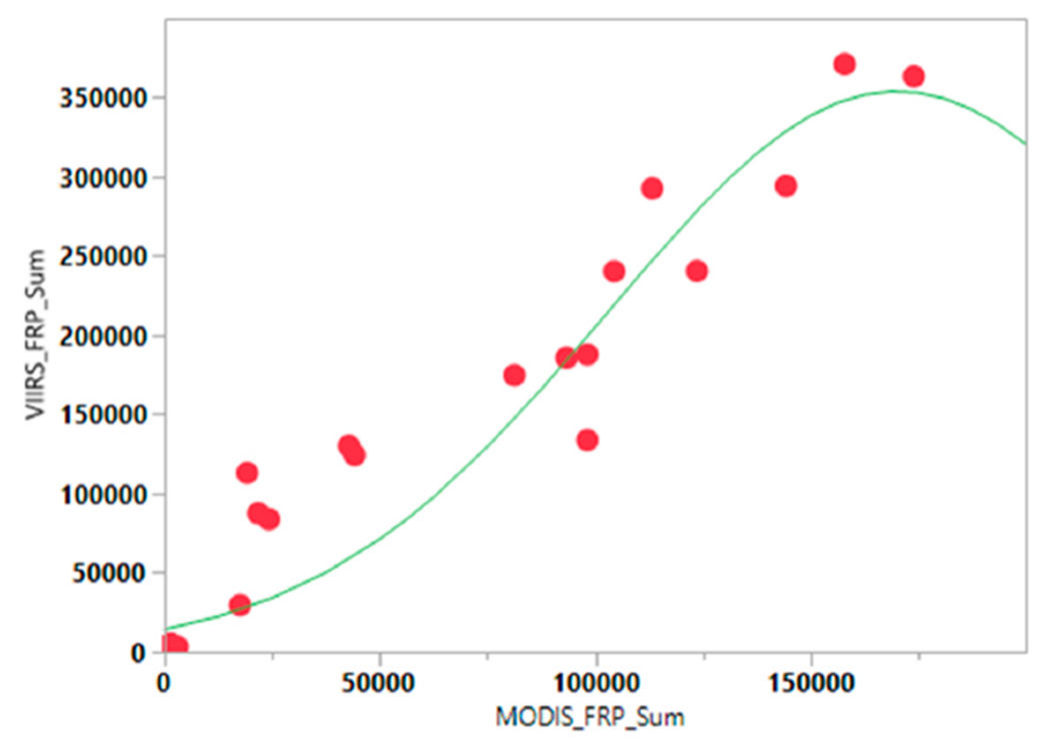
Gaussian fit of MODIS sum of FRP data and VIIRS data. The results from the fit were used to estimate TPM emissions from 2003–2016.

MODIS based FRP emissions for TPM suggested a range of 42.59–58.75 Gg in contrast to VIIRS based TPM emissions of 93.98–111.72 Gg. Of the different data used, VIIRS FRP based emissions were found to be significantly higher than the other datasets. Specifically, the TPM emissions (Gg) from VIIRS were higher by a factor of 2.25 compared to MODIS, a 1.96 factor higher than GFED, and a 1.23 factor higher than the agricultural census data-based approach (Figure 11). TPM emissions (in Gg) derived from MODIS and VIIRS FRP for different months are shown in Figure 7 and spatial variations for the peak month of November 2016 are shown in Figure 8a,b. TPM emissions data has been gridded at 5 arc minute grid intervals (10 km cells). Higher TPM emissions during November captured by VIIRS data can be seen in Figure 8b. The peak Gaussian fit results are shown in Figure 9.

**Figure 10.**
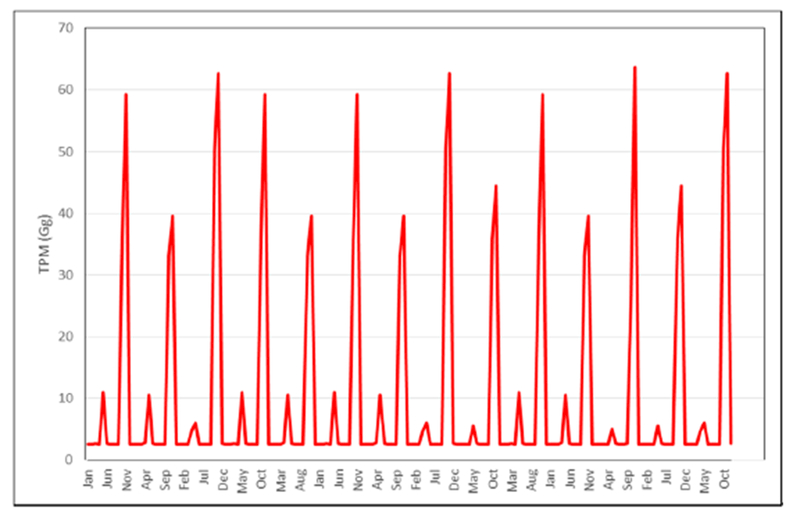
TPM emissions (2003–2016) derived from model fitting of VIIRS and MODIS data.

**Figure 11.**
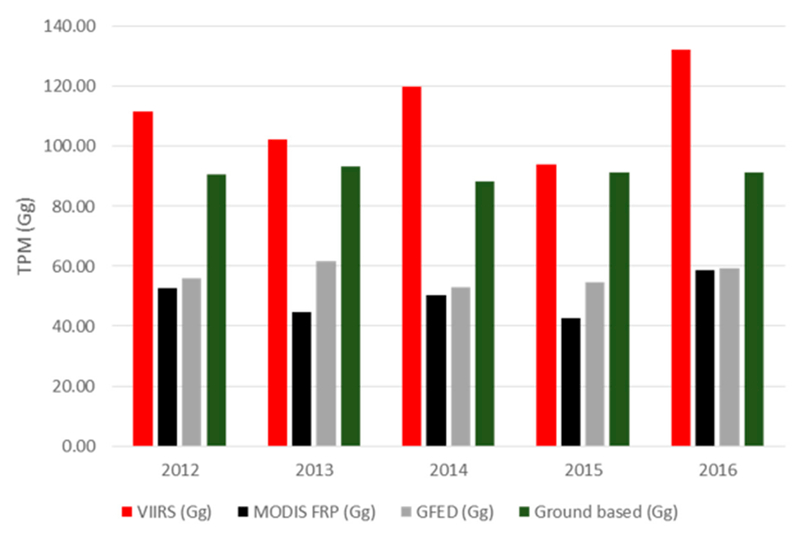
Comparison of TPM emissions from different datasets. VIIRS based emissions were found to be significantly higher than all other datasets.

The model was robust with low values of MAE (3000. The results from the model fit were used to derive the potential VIIRS TPM emissions from 2003–2016 (Figure 10) which suggested a range of 2.56–63.66 Gg TPM emissions per month, with the highest values during November of each year. Intercomparisons of TPM emissions (Gg) from different approaches is given in Figure 11. The bottom-up approach suggested TPM emissions in the range of 88.19–91.19 Gg compared to 42.0–61.71 Gg, 42.59–58.75 Gg and 93.98–111.72 Gg per year using the GFED, MODIS FRP, and VIIRS FRP based approaches, respectively (Figure 11).

## 4. Discussion

The relatively large number of fires detected by VIIRS data compared to MODIS in the agricultural landscape is consistent with the results from [37] who demonstrated an improved ability to detect ‘small fires’ using the VIIRS I-Band. They inferred that fires as small as an order of magnitude lower than the minimum detection limit of MODIS, in theory, be detected, due to the I-bands 7X smaller (nadir) pixel area. Thus, the VIIRS active fire product (VNP14IMGTDL_NRT) clearly showed much more sensitive ‘small fire’ active fire detection performance compared to the MODIS MOD14/MYD14 product in the agricultural landscape of Punjab. Possible false signals might be caused due to different illuminating conditions depending on weather and time of day. Specifically, most of the agricultural residues are thin (rice/wheat stalks) and close to the ground, sometimes exposing the bare ground which might be impacted due to specular kind of reflection impacting MIR channels, creating the possibility of false signals. Thus, strong validation is necessary to eliminate any false signals. In comparing the VIIRS and MODIS FRP, caution should be exercised while assessing the fire intensities as using the mean FRP value can produce a different solution compared to the sum of FRP. For example, a higher mean FRP from MODIS compared to VIIRS data was observed for agricultural fires in Punjab, whereas the sum of FRP was highest using VIIRS data. These results suggest a large number of smaller fires detected by VIIRS resulting in a higher sum of FRP than MODIS.

Using the IPCC based approach, [24] estimated that 620 million tons of total crop residues were burnt in India during 2008–2009, of which ~15.9% of residues were burnt in the field. Further, [24] estimated that of the different crops residues, rice straw contributed 40% of the total residue burning followed by wheat straw (22%), and sugarcane (20%). They also estimated that the crop residue burning contributed 1.21 million tons of particulate matter in 2008–2009. In the Punjab state, 56.74 million tons of residue were generated from cereal, fiber, oilseed and sugarcane crops [24]. Using these amounts, a total of 277.16 Gg TPM and 83.15 Gg PM2.5 emissions were estimated for Punjab. Compared to these estimates, our estimates suggest a range of 24–42 million tons of residue generated and a range of 49.73–112.08 Gg TPM emissions for Punjab. The discrepancies are attributed to the agricultural census data differences for different years, and conversion factors used while calculating the residue amounts as well as TPM emission factors. The low GFED based TPM emissions compared to the ground-based inventory might be due to the low biomass values for agriculture inherent in the GFED inventory [55]. Of the different datasets used, VIIRS FRP based TPM emissions were found to be higher than the MODIS, GFED and agricultural census-based estimates (Figure 10). However, we note that some satellite-based estimates currently underestimate the agricultural residue burning emissions due to small, ephemeral fires and active field management [56]. Thus, VIIRS due to its higher resolution can capture smaller fires than MODIS and in combination with the FRP based approach can yield better emission estimates.

In this study, we also compared TPM emission seasonality with other ground-based studies. For example, Reference [57] using an eight-stage size segregated mass distribution of respirable suspended particulate matter (RSPM) analysis for two wheat (summer) and three rice (winter) burning seasons, showed that fine particulate matter (PM2.5) contributing to almost 55–64% of the total RSPM, and in general smaller particles dominated during the rice crop burning during winter compared to wheat crop burning during summer. The study showed that concentration levels of PM10 and PM2.5 were higher during the winter months as compared to the summer months. Also, background concentration levels of PM10, PM2.5, and PM10–2.5 were reported to be around 97 ± 21, 57 ± 15 and 40 ± 6 μg m—3, respectively. In a separate study [58], using ground-based ambient air monitoring instruments at five different locations in and around Patiala city (29°49′−30°47′ N Latitude, 75°58′−76°54′ E Longitude), Punjab, monthly variations in suspended particulate matter (SPM), sulphur dioxide (SO_2_) and nitrogen dioxide (NO_2_) were studied. It reported that monthly average concentrations (24 h) of SPM, SO_2_ and NO_2_ varied from 100 ± 11 μg m—3 to 547 ± 152 μg m—3, 5 ± 4 μg m—3 to 55 ± 34 μg m—3 and 9 ± 5 μg m—3 to 91 ± 39 μg m—3, respectively, with the highest levels during the winter burning months (October-November) compared to the other months. Although our study does not provide specific pollutant concentration values in volume units, the seasonality of TPM emissions with higher values during winter than summer coincides with the abovementioned studies (Figures 7 and 8a,b). In particular, the sum of FRP during the winter rice residue burning months (October-December) was found to be considerably higher than the summer wheat residue burning months (Figures 4a–d and 5a). Since FRP is a significant input to emissions calculation, the results on TPM emissions are also driven by the FRP variations (Figures 7 and 8a,b) and show similar trends as the ground-based studies discussed above. In summary, compared to other approaches, the VIIRS FRP based approach was helpful for effectively addressing both the intensity, as well as the seasonal component of TPM emissions.

## 5. Conclusions

Agricultural residue burning is most prevalent in the Punjab state, northwest India. Farmers burn agricultural residues as it takes less time before planting the next crop. The summer wheat residue burning season is during April and May, and winter rice residue burning is during October and November. In this study, both, satellite as well as ground-based approaches, were used to quantify the total particulate matter (TPM) emissions and a range of emission estimates were provided. Of the different approaches, the VIIRS FRP based approach captured more emissions and the results were closer to the agricultural census-based emissions inventory. Results revealed that VIIRS detected fires were higher by a factor of 4.8 compared to MODIS Aqua and Terra sensors. Further, VIIRS detected fires were higher by a factor of 6.5 than Aqua. Thus, the VIIRS FRP based approach captured more TPM emissions than the MODIS and was closer to the emissions estimated using the agricultural census data. Specific to seasonality, the VIIRS sum of FRP was found to be higher during the winter months (rice residue burning season) than the summer (wheat residue burning season) and the values were consistently higher compared to the MODIS sum of FRP. Thus, TPM emissions were considerably higher during winter than the summer. Further, the seasonality of emissions from the VIIRS approach matched the ground-based emissions data reported in the literature. Since VIIRS data is only available from 2012 whereas MODIS Aqua is available from May 2002, we used a model fitting approach to arrive at the potential TPM emissions from VIIRS since 2002. The results suggested potential emissions of 93.98–111.72 Gg per year using the VIIRS FRP based approach. These new TPM emission outputs from Punjab can be integrated with atmospheric transport models to infer pollution in the neighboring areas, such as New Delhi where air pollution is rampant.

The current study is critical concerning air pollution research as the satellite-based FRP approach can be used to characterize TPM emissions directly. The bottom-up approach involving agricultural census data on different crops, quantifying the residue amounts and thereby emissions from the agricultural residue burning can be tedious and such data may not be available for different regions of the world. The minor discrepancies in the emissions from the satellite approach and agricultural census-based approach can be improved through improving the coefficients that directly relate FRP from fires to the mass of smoke aerosol emissions. Efforts towards strengthening research on calibrating and validating satellite FRP based smoke emissions might be the more straightforward solution for emissions quantification and more useful for pollution mapping/monitoring studies than the bottom-up agricultural census-based approach. Further, using VIIRS data compared to MODIS has significant potential as the VIIRS can capture more fires; thus it results in higher FRP and more accurate quantification of TPM emissions, as noted in this study.

We conclude that crop residue burning in Punjab, India is a management issue that needs immediate attention. Although the local government in Punjab declared the burning of crop residues in the field as illegal, and that any farmer resorting to it is liable to legal repercussions, crop residue burning continues, as evidenced by the remote sensing data. Farmers practice crop residue burning as they lack the resources to clear the residues or incorporate them into the soil through tillage machinery. In addition to implementing stringent measures on banning crop residue burning, financial aid to farmers is necessary to manage the crop residues efficiently in the field. Effective recycling of agricultural residues should be promoted by starting bioenergy plants in the region.
